# A Novel Feeder Link Handover Strategy for Backhaul in LEO Satellite Networks

**DOI:** 10.3390/s23125448

**Published:** 2023-06-08

**Authors:** Yuke Zhou, Jiang Liu, Ran Zhang, Man Ouyang, Tao Huang

**Affiliations:** 1 State Key Laboratory of Networking and Switching Technology, Beijing University of Posts and Telecommunications, Beijing 100876, China; zhouyuke@bupt.edu.cn (Y.Z.);; 2 Purple Mountain Laboratories, Nanjing 211111, China

**Keywords:** LEO satellite networks, feeder link handover, satellite-based backhaul, network flow

## Abstract

Thanks to their wide coverage and relatively low latency compared to geosynchronous satellites, Low Earth Orbit (LEO) satellite networks have been regarded as one of the most promising solutions to provide global broadband backhaul for mobile users and IoT devices. In LEO satellite networks, the frequent feeder link handover invokes unacceptable communication interruptions and affects the backhaul quality. To overcome this challenge, we propose a maximum backhaul capacity handover strategy for feeder links in LEO satellite networks. To improve the backhaul capacity, we design an available backhaul capacity ratio to jointly consider feeder link quality and the inter-satellite network in handover decisions. In addition, we introduce a service time factor and handover control factor to reduce the handover frequency. Then, we propose the handover utility function based on the designed handover factors and propose a greedy-based handover strategy. Simulation results show that the proposed strategy outperforms conventional handover strategies in backhaul capacity with low handover frequency.

## 1. Introduction

With the explosive growth of mobile users and Internet of Things (IoT) devices [1], the demands for massive global connections have never been greater [2]. However, limited by the return on investment, achieving global coverage with conventional terrestrial networks is not affordable [3]. Recently, with the development of on-board chips, laser-based Inter-Satellite Links (ISLs) [4], and launching technology, Low Earth Orbit (LEO) satellite networks have the potential to provide global high-capacity backhaul for mobile users and IoT devices [5,6]. Many companies are vigorously developing large-scale LEO communication networks to provide wide-coverage and high-capacity communication services [7]. In addition, the 3rd Generation Partnership Project (3GPP) has ongoing efforts to integrate satellite-based backhaul with 5G system in Release 18 [8].

In the LEO communication networks, each satellite serves as a mobile base station to offload traffic for the User Ends (UEs) through user links (ULs) [9] and utilize the ISLs to forward traffic to the last-hop satellite, which directly connects to the GS [10]. The ground stations (GSs) utilize the feeder links to collect backhaul traffic gathered by satellites [11] and backhaul them to the terrestrial networks. The whole transmission process is defined as the satellite-based backhaul, which is important in remote areas, hotspot areas, and emergency scenarios [8].

To improve the quality of satellite-based backhaul, many efforts have been undertaken in user accessing and routing. For example, non-orthogonal multiple access (NOMA) [12] and rate-splitting multiple access (RSMA) [13] have been introduced in satellite networks to enhance access efficiency. Meanwhile, satellite handover schemes have gained increasing attention as they can provide seamless connections for UEs [14]. Considering that networking dynamics and non-uniform traffic distribution may arouse congestion in the inter-satellite network, some load-balancing routing schemes have been proposed to provide reliable ISL-based relaying [15,16,17]. However, only some works take feeder links into account and provide satisfactory handover schemes for feeder links. Although the feeder link has more transmission capacity than the ISL, there may still be a backhaul bottleneck in the feeder link due to rain attenuation and atmospheric fading [18]. In addition, limited by geographical and political factors, GSs are generally deployed in a specific domain. Since the number of UEs far exceeds GSs, the global-distributed traffic converges to the GSs, resulting in severe congestion and heavy network load around the GSs [19]. Therefore, considering that the feeder link selection determines the last-hop satellite and the backhaul capacity, the feeder link handover is a critical issue that directly affects the performance of the satellite-based backhaul.

To solve the above issues, we propose a maximum backhaul capacity handover strategy (MBCHS) to improve the backhaul capacity by handover decision making and jointly consider the feeder links and inter-satellite network. The main contributions are summarized as follows:

- We present the LEO network scenario and formulate the feeder link handover problem to maximize the available backhaul capacity while optimizing the handover frequency.
- To solve the optimization problem, we design an available backhaul capacity ratio to estimate the available capacity of satellites for handover decisions. Then, we introduce a service time factor and handover control factor to reduce the handover frequency.
- Based on the three handover factors, we present a handover utility function for handover decisions. Then, we propose a maximum backhaul capacity handover strategy (MBCHS) based on a greedy strategy and maximum flow algorithm.
- The performance of the proposed strategy is evaluated using simulations. The results show that MBCHS outperforms the benchmarks based on maximum service time and maximum feeder link quality.

The rest of this article is organized as follows: In Section 2, we introduce the recent advances of related works. In Section 3, we formulate the network model and define the optimization problem. Then, we propose a maximum backhaul capacity handover strategy in detail in Section 4. In Section 5, simulation results are provided. Finally, we conclude the article in Section 6.

## 2. Related Works

### 2.1. Handover Schemes for User Links

Handover strategies have been investigated widely in the literature for many years. Most of them aimed at planning user-to-satellite links (USLs). Since the user service duration may be greater than the service time provided by a satellite, the user must switch its USL to another visible satellite to prevent interruption of the ongoing communication [20]. In [21], the authors investigated three common criteria, i.e., maximum service time, minimum distance, and the maximum number of free channels. The maximum service time criterion aims to reduce handover frequency and communication interruptions. In [22], the authors propose real-time dynamic update velocity-aware handover management to reduce handover frequency. The minimum distance criterion is similar to the RSS-based and maximum elevation criteria, which aim to obtain the best channel quality and improve throughput performance [23]. With the maximum idle channels criterion, the users would switch to the satellite with abundant channels to achieve load balancing and reduce the blocking rate [24].

The above three criteria mainly cater to conventional single-hop-based satellite access networks without considering the influence of ISLs. Some current research focuses on solving the handover issues in terms of the large-scale LEO networks equipped with ISLs. In [25], the authors proposed a handover strategy combined with multi-hop routing to reduce the end-to-end propagation latency. In [9], the authors proposed a congestion-aware handover scheme to achieve optimized end-to-end performance while limiting the handover frequency.

### 2.2. Handover Schemes for Feeder Links

Although scholars have carried out substantial work on the handover in satellite networks, they mainly focused on how the users maintain ongoing communication when switching the connected satellites. Efficient transmission between the LEOs and GSs is also an indispensable component for end-to-end communication [26]. In [18], multiple-input multiple-output (MIMO) technology was applied to the feeder link handover strategy to improve the achievable transmission rate of feeder links. An adaptive access selection algorithm for GS is proposed in [27]. The selection strategy of a backup access satellite and the concept of virtual destination address can reduce the routing overhead after switching.

Although several studies have been proposed to optimize the feeder link handover, they ignore that the inter-satellite network near the GSs is also a potential bottleneck. In this article, we propose a handover scheme jointly considering the feeder links and the inter-satellite network.

## 3. System Model

In this section, we present the LEO network scenario and backhaul model. Based on the network model, we propose the feeder link handover problem.

### 3.1. LEO Network Scenario

**Figure 1:**
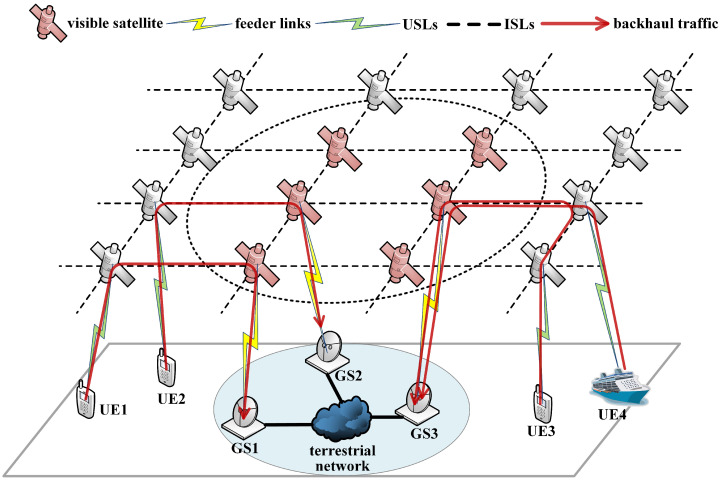
LEO communication network for backhaul services.

As illustrated in Figure 1, we present an LEO satellite network scenario where the satellites offload traffic from UEs worldwide and backhaul them to GSs through feeder links. Satellites are denoted as $S_{n}|n=1,2\ldots ,N$, where *N* represents the number of satellites in the network. Each satellite constantly moves along a circular orbit around the Earth and communicates with four adjacent satellites (two in-orbit satellites and two inter-orbit satellites) via ISLs. GSs are denoted as $G_{m}|m=1,2\ldots ,M$, where *M* represents the number of GSs. Considering the geographical and political constraints, all GSs are deployed in a limited area, as shown by the blue circle in the figure. All GSs connect to the same terrestrial network via ground links. The GS is simultaneously covered by multiple visible satellites, as shown by the dotted circle in the figure. Each GS can establish $L_{g}$ feeder links with visible satellites to offload backhaul traffic to terrestrial networks, while each satellite can connect with $L_{s}$ GSs at most. $L_{g}$ and $L_{s}$ are determined by the number of antennas equipped with satellites and GSs.

The satellite network is time-varying since the LEOs move at high speeds. The GSs must continuously switch their feeder links to suitable visible satellites to avoid interruptions of ongoing backhaul services. Virtual topology (VT) is adopted to describe the network dynamics. The VT method envisions the time-varying topology as a discrete-time network model and assumes a fixed topology in each time slot [28]. The handover decisions are only required at the beginning of each time slot t=1,2…,T.

As the red lines show in Figure 1, the feeder links and ISLs near the GSs carry a large amount of traffic since the global backhaul traffic converges to the GSs located in the limited area. Network congestion and packet loss frequently occur in the network around the GSs. Expanding the link capacity and load balancing has been widely adopted to mitigate the congestion in terrestrial networks. In satellite networks, a handover strategy based on maximizing feeder link quality can improve the channel capacity and mitigate congestion. In addition, the handover strategy affects the traffic distribution and causes the ISLs become the backhaul bottlenecks, so it is also essential to consider the ISL capacity in handover decisions.

### 3.2. Backhaul Model

In this article, we focus on solving the handover problem by jointly considering feeder links and ISLs to optimize the backhaul capacity. Then, we formulate the backhaul model related to the feeder link handover. The GSs and corresponding visible satellites are mainly considered since the backhaul traffic converges to these nodes. The visible satellite set of all GSs is denoted by V(t). The visibility $v_{n,m}\left(t\right)$ of $S_{n}$ and $G_{m}$ is defined as follows:

(1) $$v_{n,m}\left(t\right)=\left\{\begin{array}{cc} 1, & G_{m}\mathrm{is}\mathrm{visible}\mathrm{to}S_{n},\\ 0, & G_{m}\mathrm{is}\mathrm{not}\mathrm{visible}\mathrm{to}S_{n} \end{array}\right.$$

Since the satellite may be visible to more than one GS simultaneously, V(t) is expressed as

(2) $$V\left(t\right)=\{S_{n}|\sum_{m=1}^{M}v_{n,m}\left(t\right)\geq 1\}$$

During the whole communication process, GSs need to switch to a sequence of visible satellites to achieve seamless connection due to the moving of satellites. The connections between $S_{n}$ and $G_{m}$ at time slot *t* is denoted by $x_{n,m}\left(t\right)$ as follows:

(3) $$x_{n,m}\left(t\right)=\left\{\begin{array}{cc} 1, & G_{m}\mathrm{is}\mathrm{connecting}\mathrm{to}S_{n},\\ 0, & G_{m}\mathrm{is}\mathrm{not}\mathrm{connecting}\mathrm{to}S_{n} \end{array}\right.$$

In our proposed model, it is assumed that the satellites and GSs can establish multiple feeder links simultaneously. Therefore, the number of feeder links should meet the following constraints:

(4) $$\sum_{m=1}^{M}x_{n,m}\left(t\right)\leq L_{s},\;\forall n\in N$$

(5) $$\sum_{n=1}^{N}x_{n,m}\left(t\right)\leq L_{g},\;\forall m\in M$$

In our proposed model, the channel quality of the feeder link between $S_{n}$ and $G_{m}$ may be affected by the complex space environment. The channel gain $g_{n,m}\left(t\right)$ between $S_{n}$ and $G_{m}$ mainly consists of the path loss, atmospheric fading, and Rician small-scale fading [29], are is expressed as follows:

(6) $$g_{n,m}\left(t\right)={\left(\frac{c}{4\pi vd_{n,m}\left(t\right)}\right)}^{2}\cdot 10^{\frac{3\chi d_{n,m}\left(t\right)}{10h}}\cdot Ri$$

where ${\left(\frac{c}{4\pi vd_{n,m}\left(t\right)}\right)}^{2}$ is path loss, $10^{\frac{3\chi d_{n,m}\left(t\right)}{10h}}$ is atmospheric fading, Ri is Rician small-scale fading. *c* and fc are the speed of light and the carrier frequency, χ is attenuation through the clouds and rain in dB/km, and *h* is the altitude of satellites. The above four parameters and Ri can be approximated as constants. Therefore, $d_{n,m}\left(t\right)$ is the only parameter varying with time, which represents the distance between $S_{n}$ and $G_{m}$.

The available feeder link capacity from $S_{n}$ to $U_{m}$ at time *t* is expressed as follows:

(7) $$C_{n,m}^{g}\left(t\right)=B_{g}\log_{2}\left(1+\frac{g_{n,m}\left(t\right)P_{t}g_{t}g_{s}}{\sigma^{2}}\right)$$

where $P_{t}$ is the transmission power of the satellite, $g_{t}$ is the antenna gain of the transmitter, $g_{s}$ is the antenna gain of the receiver, $\sigma^{2}$ the noise power at GS, and $B_{g}$ is the bandwidth allocated to the feeder link. The channels between satellites are regarded as ideal channels regardless of attenuation. Therefore, the available channel capacity of ISLs is assumed as a constant $C^{s}$.

The object of feeder link handover is to optimize the backhaul capacity, defined as the maximum backhaul flow in the graph. To formulate the traffic flow, a binary indicator $l_{i,j}$ is used to indicate whether $S_{i}$ links to $S_{j}$ as follows:

(8) $$l_{i,j}=\left\{\begin{array}{cc} 1, & S_{i}\;\mathrm{links}\;\mathrm{to}\;S_{j},i\neq j,\\ 0, & S_{i}\;\mathrm{does}\;\mathrm{not}\;\mathrm{link}\;\mathrm{to}\;S_{j},i\neq j \end{array}\right.$$

The traffic flow from $S_{i}$ to $S_{j}$ is denoted as $f_{i,j}^{s}\left(t\right)$. For each satellite $S_{n}$ in the visible satellite set, it receives traffic from its adjacent satellites and connecting UEs, which is denoted by $f_{n}^{in}\left(t\right)$ as follows:

(9) $$f_{n}^{in}\left(t\right)=\sum_{j=1,j\neq n}^{N}l_{j,n}f_{j,n}^{\mathrm{isl}}\left(t\right)+f_{n}^{u}\left(t\right),\;\forall S_{n}\in V\left(t\right)$$

where $f_{n}^{u}\left(t\right)$ is the overall traffic flow of the ULs of $S_{n}$. The overall capacity of the ULs is assumed to be $C^{u}$ at most. Accordingly, each satellite needs to transmit traffic to its adjacent satellites and its connecting GSs, which is denoted by $f_{n}^{out}\left(t\right)$ as follows:

(10) $$f_{n}^{out}\left(t\right)=\sum_{j=1,j\neq n}^{N}l_{n,j}f_{n,j}\left(t\right)+\sum_{m=1}^{M}x_{n,m}\left(t\right)f_{n,m}^{g}\left(t\right),\;\forall S_{n}\in V\left(t\right)$$

where $f_{n,m}^{g}\left(t\right)$ is the traffic flow from $S_{n}$ to $G_{m}$.

Based on Equations (3) and (10), the available backhaul capacity in the time slot *t* is defined as the overall traffic flow capacity of all feeder links as follows:

(11) $$\sum_{m=1}^{M}\sum_{n=1}^{N}x_{n,m}\left(t\right)f_{n,m}^{g}\left(t\right)$$

### 3.3. Problem Formulation

In the system, massive global traffic needs to be backhauled to the terrestrial network through the satellite network and a few GSs, arousing network congestion in the feeder links and surrounding the inter-satellite network. Therefore, in this article, we aim to maximize the available backhaul capacity by optimizing the feeder link handover problem, which is defined as follows:

(12) $$\sum_{t=1}^{T}\sum_{m=1}^{M}\sum_{n=1}^{N}x_{n,m}\left(t\right)f_{n,m}^{g}\left(t\right)$$

Meanwhile, maximizing the instantaneous throughput at each time slot may arouse frequent handover, interrupting ongoing communications and producing massive routing overhead. The handover cost is introduced to reduce the handover frequency as follows:

(13) $$h_{n,m}\left(t\right)=\left\{\begin{array}{cc} 1, & x_{n,m}\left(t\right)=1,\;x_{n,m}\left(t-1\right)=0\\ 0, & x_{n,m}\left(t\right)=x_{n,m}\left(t-1\right) \end{array}\right.$$

where the handover cost is zero if the feeder link does not switch in time slot *t*. Therefore, the ultimate optimization that balances the long-term backhaul performance and the handover frequency is formulated as follows:

(14) $$\begin{array}{cc} \mathrm{OP}: & \;\max\sum_{t=1}^{T}\sum_{m=1}^{M}\sum_{n=1}^{N}x_{n,m}\left(t\right)\left(f_{n,m}^{g}\left(t\right)-\gamma h_{n,m}\left(t\right)\right) \end{array}$$

(14a) $$\begin{array}{cc} s.t. & \;\;x_{n,m}\left(t\right)\in \{0,1\},\;\;\;\forall n\in N,m\in M,t\in T \end{array}$$

(14b) $$\begin{array}{c} \;\sum_{m=1}^{M}x_{n,m}\left(t\right)\leq L_{s},\;\;\forall n\in N,t\in T \end{array}$$

(14c) $$\begin{array}{cc} \sum_{n=1}^{N}x_{n,m}\left(t\right)\leq L_{g}, & \;\;\forall m\in M,t\in T \end{array}$$

(14d) $$\begin{array}{cc} f_{n}^{in}\left(t\right)=f_{n}^{out}\left(t\right), & \;\;\;\forall n\in N,t\in T \end{array}$$

(14e) $$\begin{array}{cc} 0\leq f_{n,m}^{g}\left(t\right)\leq C_{n,m}^{g}\left(t\right), & \;\forall n\in N,m\in M,t\in T \end{array}$$

(14f) $$\begin{array}{cc} 0\leq f_{i,j}^{\mathrm{isl}}\left(t\right)\leq C^{s}, & \;\;\;\forall i,j\in N,t\in T \end{array}$$

(14g) $$\begin{array}{cc} 0\leq f_{n}^{u}\left(t\right)\leq C^{u}, & \;\;\forall n\in N,t\in T \end{array}$$

where γ represents the handover’s influence on the backhaul. The constraint (14a) means that $x_{n,m}\left(t\right)$ is a binary indicator representing whether $S_{n}$ establishes a link with $U_{m}$ or not at time slot *t*. The constraints (14b) and (14c) limit the number of established links for each node. The traffic of a satellite needs to follow the constraint (14d). The constraints (14e)–(14g) indicate that the backhaul traffic in links cannot exceed the available channel capacity.

## 4. Problem Analysis and Strategy Design

The proposed optimization problem is a mixed-integer programming problem, and the complexity of obtaining the optimal solution is NP-hard. To solve this optimization, we analyze the optimization problem and design an available backhaul capacity ratio to estimate the available capacity of satellites in the handover decisions. Then, we propose the maximum backhaul capacity handover strategy based on the available backhaul capacity ratio.

### 4.1. Problem Analysis

As Equation (14) shows, the optimization comprises handover decision variables and traffic distribution variables. Thereby, the optimization is decomposed into the feeder link handover problem and maximum flow problem. The handover decision variables are determined by solving the handover problem. Then, based on the network topology and link constraints, the maximum flow problem is solved to determine the available backhaul capacity in the satellite network. Note that the order of these two sub-problems cannot be reversed since the network topology depends on the handover results.

The Ford–Fulkerson augmenting flow algorithm is widely used to find the maximum flow from a source to a sink in a directed graph. The classic algorithms include Edmonds–Karp and Dinic. These algorithms utilize the BFS-based method to find the augmenting paths to calculate the maximum flow. Although the solution for the maximum flow problem is mature, the maximum flow problem in this optimization problem is quite different. As shown in Equation (10), the outgoing traffic of $S_{n}$ is composed of the traffic it transmits to neighboring satellites and the traffic to the GSs. The latter is related to the handover decision variables, so the handover strategy would affect the maximum flow calculation.

Therefore, it is essential to explore the handover’s effects on the maximum flow in the handover decisions. We propose an available backhaul capacity ratio to predict the available backhaul flow. Unlike the achievable transmission rate based on the channel quality represented in [23], the available backhaul capacity ratio is based on the combination of feeder link capacity and inter-satellite network capacity. The available feeder link capacity is expressed by the normalized channel capacity as follows:

(15) $$r_{n,m}^{g}\left(t\right)=\frac{C_{n,m}^{g}\left(t\right)}{C_{\max}^{g}}$$

where $C_{\max}^{g}$ is a constant representing the maximum channel capacity of feeder links.

In addition, the available backhaul capacity also depends on the available satellite capacity, which is related to the traffic the satellite can receive from its neighboring nodes. The available satellite capacity is defined as the sum of the USLs’ remaining capacity and the ISLs’ remaining capacity. Since the traffic generally transmits through multiple satellites, the available capacity of these satellites should be updated after each handover decision.

The calculation process of the available satellite capacity is described as follows: (1) At the beginning of each time slot *t*, the available satellite capacity of each visible satellite is initialized to the sum capacity of its adjacent ISLs and USLs. (2) Then, the handover decisions are figured out in sequence. After each handover decision, the available satellite capacity of each visible satellite is updated based on the hops to the connecting satellite and the available feeder link capacity of the connecting satellite.

**Figure 2:**
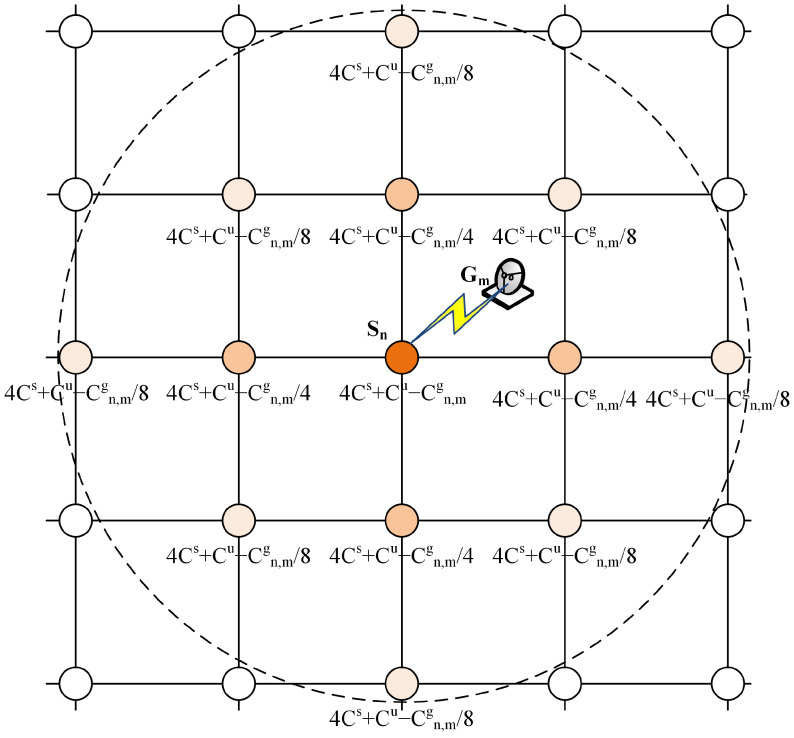
Schematic diagram of updating the available satellite capacity.

Figure 2 illustrates the capacity update strategy. The GS $G_{m}$ establishes a feeder link with the satellite $S_{n}$, whose available link capacity is $C^{g}n,m$. The available satellite capacity of the directed connected satellite $S_{n}$ would decrease $C^{g}n,m$. The available satellite capacity of the adjacent satellites of $S_{n}$ would decrease $\frac{C^{g}n,m}{4}$. Moreover, there are eight satellites that are two hops away from $S_{n}$, so their available satellite capacity would decrease $\frac{C^{g}n,m}{8}$. In the scenario, the available satellite capacity of a satellite that is *h* hops away from the access satellite would decrease $\frac{C^{g}n,m}{4h}$. The shade of the satellite nodes shown in the figure represents the influence of the feeder link on the satellites’ capacity. When completing a handover decision, the available satellite capacity is expressed as follows:

(16) $$C_{i}^{s}\left(t\right)=4C^{\mathrm{isl}}+C^{u}-\sum_{m}^{M}\left(\sum_{n\neq i}^{N}x_{n,m}\left(t\right)\frac{C_{n,m}^{g}\left(t\right)}{4h_{n,i}}+x_{i,m}\left(t\right)C_{i,m}^{g}\left(t\right)\right)$$

where $4C^{\mathrm{isl}}+C^{u}$ is the limit of the available capacity of the satellite, and the remaining is the overall influence of all feeder links on the available satellite capacity.

Next, the available satellite capacity must be normalized to the available satellite capacity ratio $r^{s}n,m\left(t\right)$. When $C_{i}^{s}\left(t\right)$ is large, $r^{s}n,m\left(t\right)$ should keep close to one since the feeder link is more likely to be the bottleneck. When $C_{i}^{s}\left(t\right)$ is small, the ISLs are more likely to be the bottleneck so $r^{s}n,m\left(t\right)$ must accurately reflect the subtle changes in $C_{i}^{s}\left(t\right)$. Therefore, a sigmoid function is used for normalization as follows:

(17) $$r_{n,m}^{s}\left(t\right)=\frac{1}{1+e^{-\alpha (\frac{C_{n}^{s}\left(t\right)}{C_{\max}^{g}}-\beta )}}$$

where β is the offset and α represents the gradient. This normalization method makes $r_{n,m}^{s}\left(t\right)$ close to one when the available satellite capacity exceeds the available feeder link capacity and close to zero when the available satellite capacity is small. $r_{n,m}^{s}\left(t\right)$ would change significantly if the available satellite capacity ranged from zero to the upper limits of the available feeder link capacity.

Considering that the backhaul capacity depends on the status of the ISLs and the feeder links, the available backhaul capacity ratio is expressed as follows:

(18) $$r_{n,m}^{b}\left(t\right)=r_{n,m}^{s}\left(t\right)r_{n,m}^{g}\left(t\right)$$

The available backhaul capacity ratio can estimate the maximum flow performance achieved by the feeder link handover and improve the backhaul capacity performance in the optimization. To reduce the handover frequency, we introduce the service time factor and handover control factor. The service time factor facilitates the feeder links switching to a satellite with a larger visible time, which can be calculated based on the ephemeris and the locations. The service time factor is expressed as follows:

(19) $$r_{n,m}^{t}\left(t\right)=\frac{D_{n,m}\left(t\right)}{D_{\max}}$$

where $D_{n,m}\left(t\right)$ is the remaining time that $S_{n}$ is visible to $G_{m}$. $D_{\max}$ is the upper limit of the remaining service time.

If a UE aims at maximizing the instantaneous throughput at each time slot, this UE may change its NT-BS selection at every time slot, which results in frequent handovers. The handover control factor is introduced to avoid frequent handovers in a greedy strategy. It is expressed as follows:

(20) $$r_{n,m}^{h}\left(t\right)=1-h_{n,m}\left(t\right)$$

Based on the proposed three handover factors, we propose a handover utility function for handover decisions expressed as follows:

(21) $$W_{n,m}\left(t\right)=w_{1}r_{n,m}^{b}\left(t\right)+w_{2}r_{n,m}^{t}\left(t\right)+w_{3}r_{n,m}^{h}\left(t\right)$$

where $w_{1}$, $w_{2}$, and $w_{3}$ represent the weights of each handover factor with the following constraint:

(22) $$w_{1}+w_{2}+w_{3}=1$$

### 4.2. Maximum Backhaul Capacity Handover Algorithm

Based on the handover utility function, we propose a maximum backhaul capacity handover algorithm, as shown in Algorithm 1. The algorithm is divided into two steps: handover decision making and maximum flow calculation.

**Figure 3:**
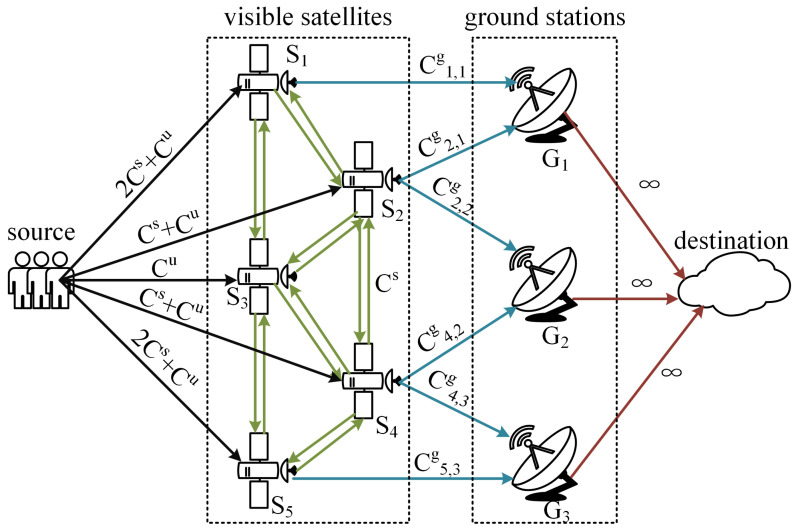
The graph for maximum flow problem.

(1) Handover decision making: First, the visible satellites of each GS are figured out and added to the visible satellite set. In addition, the remaining service time and handover control factor between each GS and its visible satellites are calculated. The handover decision variables and available satellite capacity are initialized. Then, the handover decisions are looped $L_{g}M$ times, which is the maximum number of the feeder links. In each loop, the available backhaul capacity is calculated to obtain the handover utility. Then, the $W_{n,m}\left(t\right)$ with maximum value is selected as the handover result in this loop, and the available satellite capacity of all visible satellites is updated.

(2) Maximum flow calculation: First, the graph must be built for the maximum flow calculation, as shown in Figure 3. The terrestrial network is set as the sink of the graph, since the backhaul traffic would converge to the same terrestrial network. *M* edges with infinite capacity are added to the graph to represent the links between GSs and the sink as the red directed arrows show in the figure. In this scenario, the visible satellites can collect backhaul traffic from their USLs or their adjacent invisible satellites by ISLs. These sources are aggregated into a source node to facilitate the computation. |V(t)| edges are added between the source and the visible satellites, as the black directed arrows show in the figure. The edge’s capacity is the sum capacity of USLs and ISLs connecting with the invisible satellites. In addition, the edges with capacity $C^{s}$ are added to represent the ISLs between visible satellites. Then, the edges of feeder links are added based on the handover results of Step 1. Finally, the Edmonds–Karp algorithm is used to calculate the maximum flow based on the constructed graph.
**Algorithm 1** Maximum backhaul capacity handover strategy1:**Input:** Network Topology, termination time slot *T*, weights of utility function $w_{1},w_{2},w_{3}$, parameters of sigmoid function α,β.2:**for**t=0 to *T* **do**3: **(1) Handover decision:**4: Compute the visible satellites for $U_{m}$, and put the visible satellites into visible satellite set V(t).5: Compute the remaining service time $r_{n,m}^{t}\left(t\right)$ and the handover control factor $r_{n,m}^{h}\left(t\right)$ between $U_{m}$ and $S_{n}$.6: Initial the available satellite capacity $C_{i}^{s}\left(t\right)=4C^{s}+C^{u}$.7: Initial the handover decision variables $x_{n,m}\left(t\right)=0$.8: **for** k=0 to $L_{g}M$ **do**9:  **for** m=1 to *M* **do**10:   **if** $\sum_{n=1}^{N}x_{n,m}\left(t\right)<L_{g}$ **then**11:    **for** $S_{n}$ in V(t) **do**12:     **if** $\sum_{m=1}^{M}x_{n,m}\left(t\right)<L_{s}$ **then**13:      Compute the normalized value $r_{n,m}^{b}\left(t\right)$ and get the utility $W_{n,m}\left(t\right)$ as Equation (21).14:     **end if**15:    **end for**16:   **end if**17:  **end for**18:  Select the $W_{n,m}\left(t\right)$ with maximum value and let $x_{n,m}\left(t\right)=1$.19:  Update the available satellite capacity for all visible satellites.20: **end for**21: **Output:** the handover frequecy $\sum_{m=1}^{M}\sum_{n=1}^{N}x_{n,m}\left(t\right)$.22: **(2) Maximum flow computation:**23: Build graph *G* based on the handover decisions and link capacity.24: **while** True **do**25:  Build the augmented graph $G_{a}$. Compute the path *p* from *s* to *d* with minimum hops.26:  **if** p==null **then**27:   **break**28:  **end if**29:  Find the edge with minimum remaining capacity $f_{min}$ in the path *p*.30:  Update the flow of all edges in the path.31: **end while**32: **Output:** the maximum flow $\sum_{m=1}^{M}\sum_{n=1}^{N}x_{n,m}\left(t\right)f_{n,m}^{g}\left(t\right)$.33:**end for**

### 4.3. Handover Workflow

**Figure 4:**
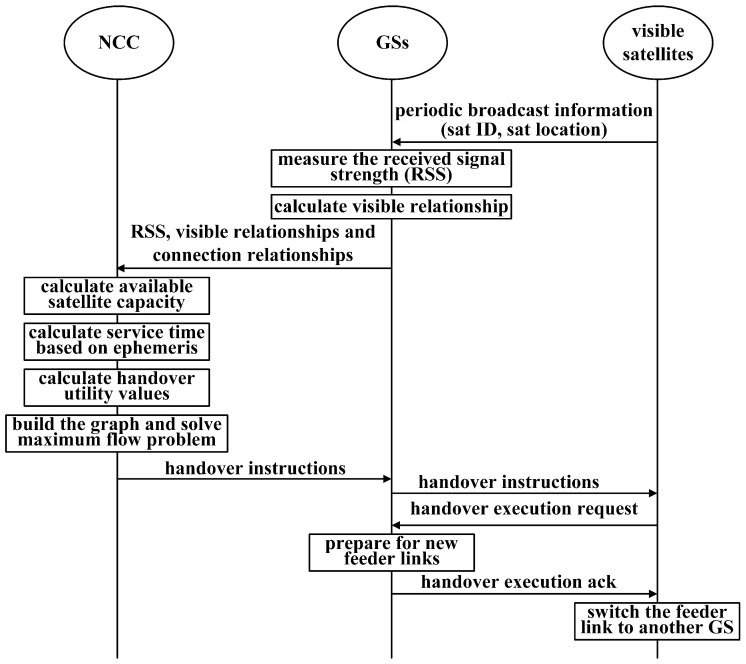
Flow chart of the feeder link handover procedure.

Figure 4 illustrates the flow chart of the feeder link handover procedure. At first, the GSs monitor the periodic broadcast information sent by visible satellites and measure the received signal strength (RSS). Then, the GSs calculate the visible relationships and send the required handover information to the NCC, including RSS, visible relationships, and connection relationships. The NCC calculates the remaining service time and available satellite capacity based on Equation (18) for all satellites. Based on these values, the NCC figures out the handover utility values based on Equation (21) and builds a graph to solve the maximum flow problem with Algorithm 1. Then, the NCC sends the handover instructions to the corresponding satellites and GSs. The visible satellites release the old links and build new links to the GSs. At this step, a round of feeder link handover decisions is finished.

## 5. Simulation and Analysis

In this section, we evaluate the performance of the proposed handover strategy and compare it with other handover schemes.

### 5.1. Simulation Settings

We chose the Walker delta satellite constellations with an orbital altitude of 1000 km and an orbital inclination of $54^{\circ }$. The GSs are randomly located in the domain from $90^{\circ }E$ to $120^{\circ }E$ and from $20^{\circ }N$ to $45^{\circ }N$. The minimum elevation angle of the GS antennas is $5^{\circ }$. The number of satellites, the number of GSs, and the weights of the utility function are set as variables. The parameters of sigmoid function α and β are determined based on prior knowledge. Other detailed parameters of the simulation are presented in Table 1.

To verify the effectiveness and advantages of the proposed scheme, we selected the Maximum Service Time Handover Strategy (MSTS) [22] and the Maximum GSL (ground-to-satellite) Capacity Handover Strategy (MGCS) as performance comparison benchmarks. The GSL capacity is calculated based on Equation (7).

### 5.2. Simulation Results and Analysis

First, we evaluated the backhaul capacity and handover frequency performance with diverse satellite and GS scales. The backhaul capacity is defined as the maximum flow of the network graph in each time slot. The handover frequency is equal to the average handover times of each GSs in an orbital period (around 6000 s). In these experiments, the weights of the utility function were set as $w_{1}=0.64$, $w_{2}=0.16$, and $w_{3}=0.2$.

**Figure 5:**
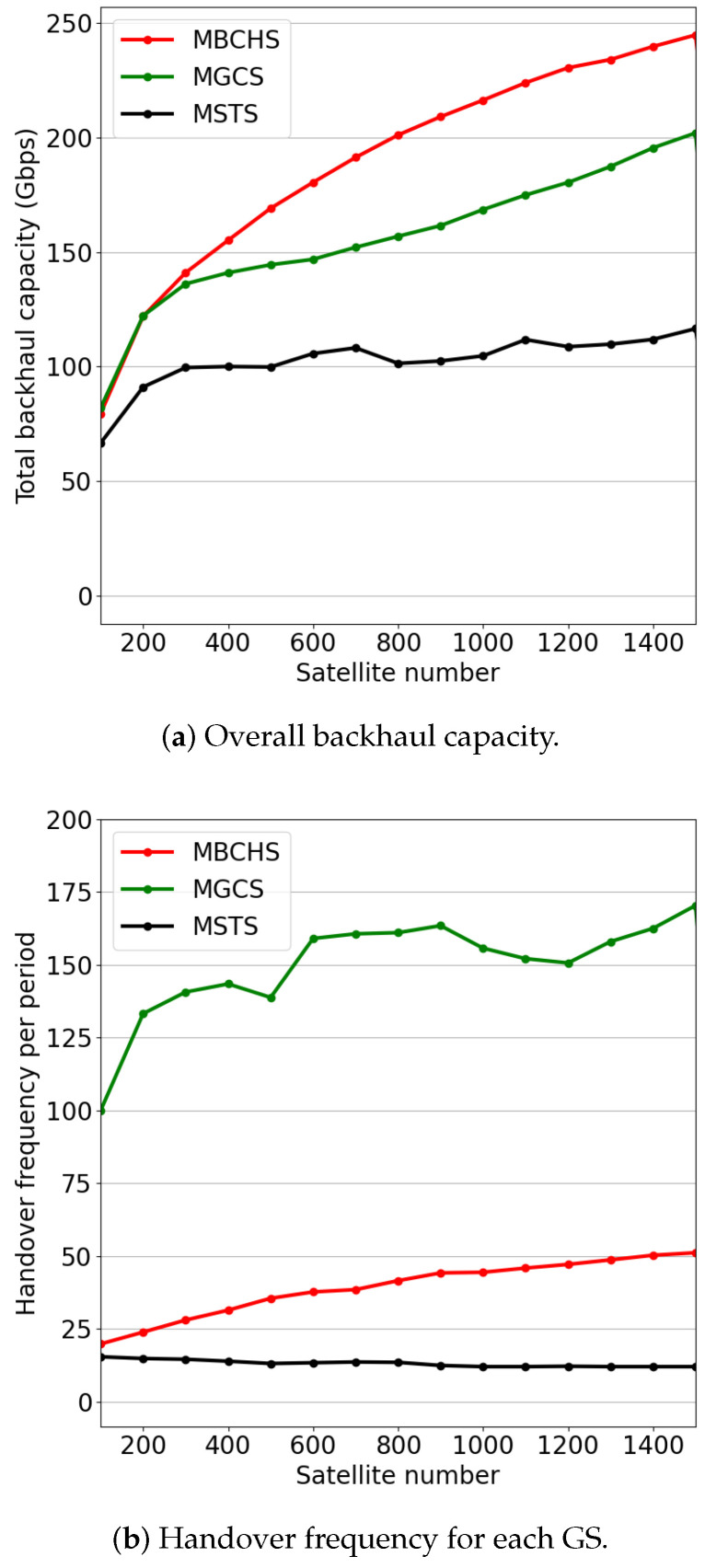
The performance comparison with different numbers of satellites.

Figure 5 proves the backhaul capacity and handover frequency performance with different constellation scales. The number of GSs is set to M=15. The satellite number varies from 100 to 1500. As shown in Figure 5a, the backhaul capacity increases with the increase in satellite number. MBCHS and MGCS outperform the MSTS in backhaul capacity. As the satellite number is less than 300, the performance of MBCHS is similar to that of MGCS. The reason is that only a few satellites can be considered in handover decisions as the constellation density is small. Thus, the handover results of these strategies are highly similar. As the satellite number increases from 300 to 1500, MBCHS outperforms MGCS, which indicates that the proposed handover strategy can effectively improve the backhaul capacity in a large-scale satellite network. As shown in Figure 5b, the handover frequency of MBCHS is slightly higher than that of MSTS since MSTS focuses on minimizing the handover frequency. Meanwhile, the handover frequency of MBCHS is much lower than that of MGCS, which also aims at maximizing the network capacity.

**Figure 6:**
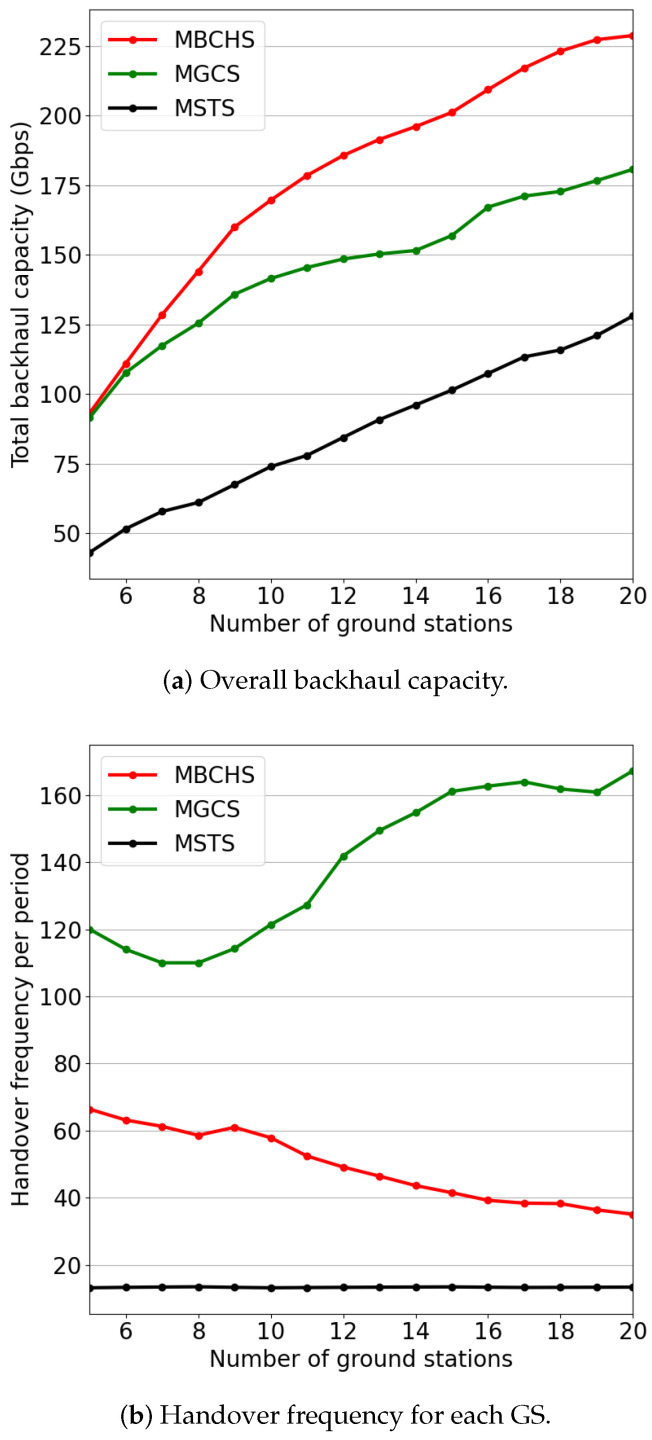
The performance comparison with different numbers of GSs.

Figure 6 indicates the backhaul capacity and handover frequency performance with different numbers of GSs varying from 5 to 20. The number of satellites is set to N=800. As shown in Figure 6a, the backhaul capacity of the three schemes increases with the increase in GS number because the number of feeder links for the backhaul service increases. Obviously, MBCHS outperforms the benchmarks in backhaul capacity while the handover frequency is moderate. As shown in Figure 6b, the handover frequency of MBCHS is between these two benchmarks. Meanwhile, the handover frequency of MBCHS gradually approaches MSTS while the GS number increases.

**Figure 7:**
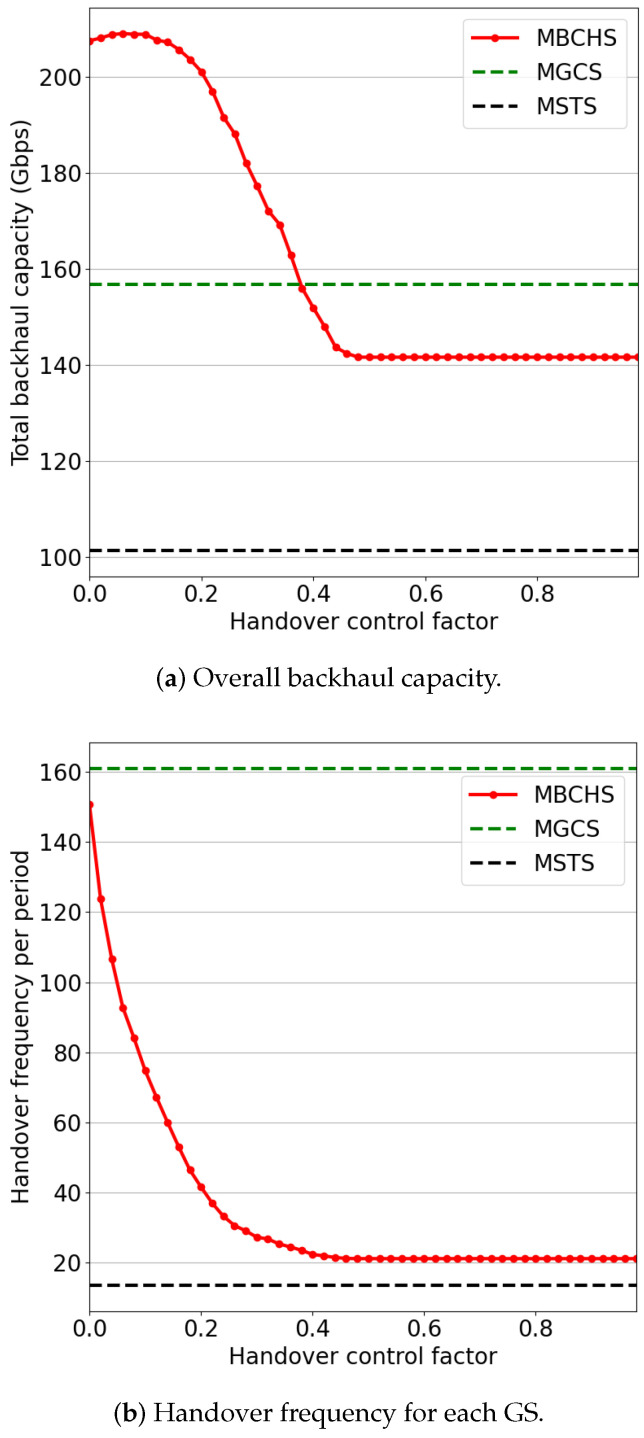
The performance comparison with different handover control factors.

To explore the optimal weight setting of the utility function, we also evaluated the performance with different weights of the handover control factor and service time factor. Figure 7 shows the simulation results with $w_{3}$, while $w_{1}=0.8*\left(1-w_{3}\right)$ and $w_{2}=0.2*\left(1-w_{3}\right)$. As the weight varies from 0 to 0.2, the handover frequency drops rapidly while the backhaul capacity changes slightly. The backhaul capacity also drops rapidly as the weight varies from 0.2 to 0.4. When the weight is greater than 0.4, the backhaul capacity and handover frequency vary slightly. Thus, selecting a small weight for the handover control factor can significantly reduce the handover frequency while ensuring the backhaul capacity performance.

**Figure 8:**
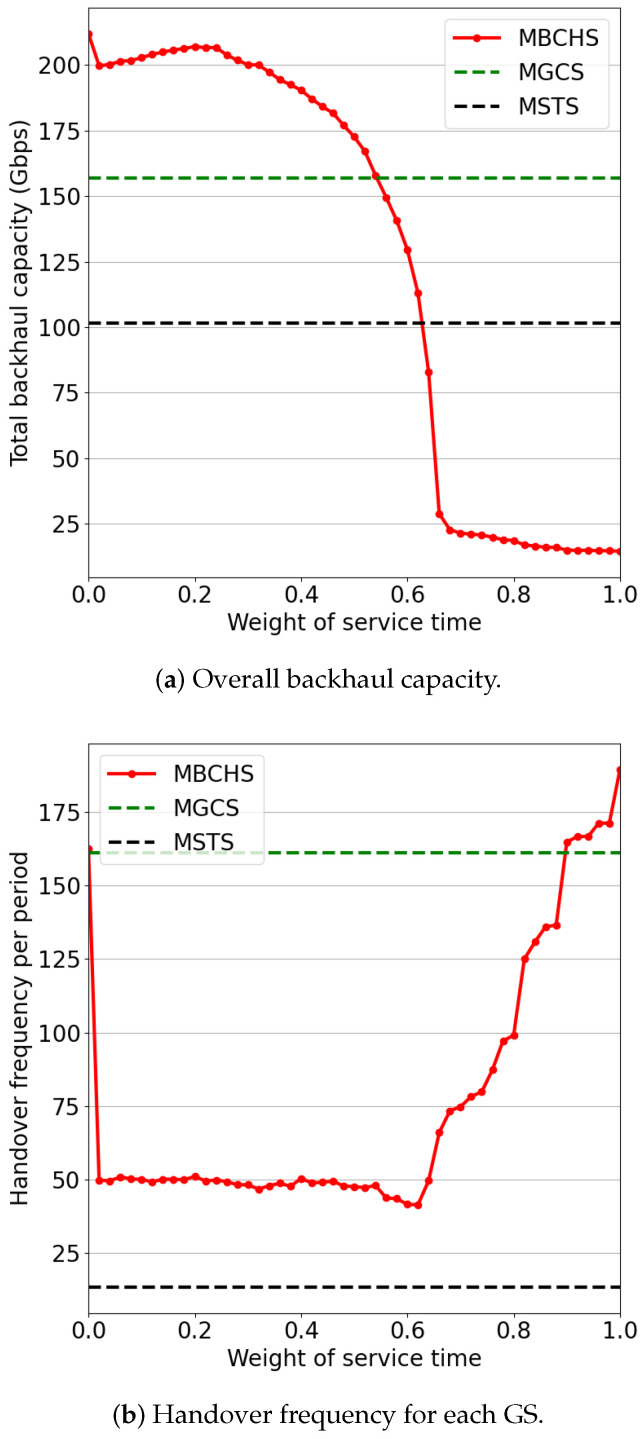
The performance comparison with different weights of service time.

Figure 8 shows the simulation results with different weights of service time factor while $w_{1}=0.8*\left(1-w_{2}\right)$ and $w_{3}=0.2*\left(1-w_{2}\right)$. As shown in Figure 8a, a small weight can significantly reduce the handover frequency. The reason is that there are many visible satellites with similar weights in the handover decision-making process. Thus, the service time factor can also play an important role, even if it is small. As the weight varies from 0 to 0.4, the backhaul capacity and handover frequency both change slightly. When the weight is greater than 0.6, the backhaul capacity drops rapidly while the handover frequency increases rapidly. The reason is that each feeder link tends to switch to another satellite with a larger service time at each time slot when the weight is greater than 0.6. These satellites with large service times are located at the margin of the GS’s coverage area and have poor channel quality. Thus, the backhaul capacity performance is extremely low. Therefore, the weight of the service time factor should be set close to 0.

**Figure 9:**
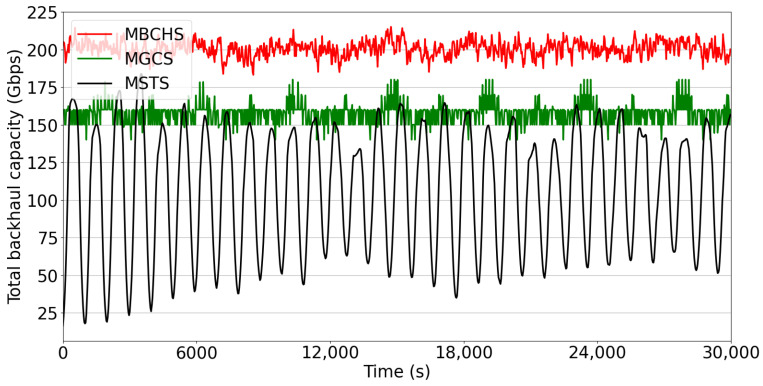
The overall backhaul capacity performance.

Finally, the backhaul capacity versus time slots is presented in Figure 9. The number of satellites is set to N=800 and the number of GSs is set to M=15. The weights of the utility function are set as $w_{1}=0.64$, $w_{2}=0.16$, and $w_{3}=0.2$. Obviously, MBCHS outperforms both benchmarks at all times. Compared with MGCS, which also aims at maximizing network capacity, the proposed strategy improves the backhaul capacity performance by 25%. The average backhaul capacity of the proposed algorithm is almost 200 Gbps, while the average backhaul capacity of MGCS is almost 159 Gbps. Note that the curve of MSTS varies greatly with time. The reason is that the handover occurs only when the satellite is out of sight and the feeder link connections are relatively stable for a long duration. As the satellite moves periodically at a circular orbit, the backhaul capacity would change as the propagation distance of the feeder link changes to small and then to large.

## 6. Conclusions

LEO satellite networks have been regarded as one of the most promising methods to provide global broadband backhaul in the 6G era. In LEO satellite networks, the frequent feeder link handover invokes unacceptable communication interruptions and affects the backhaul quality. To overcome this challenge, we propose a novel feeder link handover strategy for backhaul in LEO satellite networks to improve the backhaul quality. We formulate the feeder link handover problem to maximize the available backhaul capacity while optimizing the handover frequency. To solve the optimization problem, we design an available backhaul capacity ratio to jointly consider feeder link quality and inter-satellite networks in handover decisions. In addition, we introduce the service time factor and handover control factor to reduce the handover frequency. Based on these handover factors, we propose a handover utility function and a maximum backhaul capacity handover strategy to optimize the long-term backhaul capacity while avoiding frequent handovers. The simulation results have proved that our proposed strategy effectively outperforms the state-of-the-art schemes in terms of the backhaul capacity and handover frequency.

The proposed handover strategy mainly aims to increase the upper limit of network backhaul capacity. However, advanced network routing and traffic engineering technologies are critical for implementing backhaul optimization in practical scenarios. In future works, we intend to investigate the integration of routing and feeder link handover to realize a more comprehensive backhaul optimization scheme.

## Figures and Tables

**Table 1:** Simulation parameters.

Parameters	Value
Number of satellites	100∼1500
Orbital altitude	1000 km
Orbital inclination	$54^{\circ }$
Number of GSs	5∼20
Locations of GSs	$90^{\circ }$ E∼$120^{\circ }$ E, $20^{\circ }$ N∼$45^{\circ }$ N
Minimum elevation of antennas	$5^{\circ }$
Capacity of ISLs/USLs	5 Gbps/2 Gbps
Carrier frequency	20 GHz
Bandwidth of feeder links	2 GHz
Other parameters in Equation (7)	χ = −2, Ri = 1, $P_{t}g_{t}g_{s}$ = 90 dB
Parameters of sigmoid function	α = 4, β = 0.5

## Data Availability

The data presented in this study are available on request from the corresponding author.
